# Exploring the optimal indicator of short‐term peridiagnosis weight dynamics to predict cancer survival: A multicentre cohort study

**DOI:** 10.1002/jcsm.13467

**Published:** 2024-04-21

**Authors:** Liangyu Yin, Ling Zhang, Long Li, Ming Liu, Jin Zheng, Aiguo Xu, Quanjun Lyu, Yongdong Feng, Zengqing Guo, Hu Ma, Jipeng Li, Zhikang Chen, Hui Wang, Zengning Li, Chunling Zhou, Xi Gao, Min Weng, Qinghua Yao, Wei Li, Tao Li, Hanping Shi, Hongxia Xu

**Affiliations:** ^1^ Department of Clinical Nutrition Daping Hospital, Army Medical University (Third Military Medical University) Chongqing China; ^2^ Department of Nephrology, the Key Laboratory for the Prevention and Treatment of Chronic Kidney Disease of Chongqing, Chongqing Clinical Research Center of Kidney and Urology Diseases Xinqiao Hospital, Army Medical University (Third Military Medical University) Chongqing China; ^3^ Department of General Surgery The Second Affiliated Hospital of Harbin Medical University Harbin China; ^4^ Department of Traditional Chinese Medicine Tangdu Hospital, Air Force Medical University (The Fourth Military Medical University) Xi'an China; ^5^ Department of Respiratory and Critical Care Medicine The First Affiliated Hospital of Zhengzhou University Zhengzhou China; ^6^ Department of Nutrition The First Affiliated Hospital of Zhengzhou University Zhengzhou China; ^7^ Department of GI Cancer Research Institute Tongji Hospital, Tongji Medical College, Huazhong University of Science and Technology Wuhan China; ^8^ Department of Medical Oncology Fujian Cancer Hospital, Fujian Medical University Cancer Hospital Fuzhou China; ^9^ Department of Oncology The Affiliated Hospital of Zunyi Medical University Zunyi China; ^10^ Department of Experimental Surgery Xijing Hospital, Fourth Military Medical University Xi'an China; ^11^ Department of Colorectal and Anus Surgery Xiangya Hospital of Central South University Changsha China; ^12^ Department of Oncology The People's Hospital of Dujiangyan Dujiangyan China; ^13^ Department of Clinical Nutrition The First Hospital of Hebei Medical University Shijiazhuang China; ^14^ Department of Clinical Nutrition The Fourth Affiliated Hospital of Harbin Medical University Harbin China; ^15^ Department of Oncology Affiliated Hospital of North Sichuan Medical College Nanchong China; ^16^ Department of Clinical Nutrition The First Affiliated Hospital of Kunming Medical University Kunming China; ^17^ Department of Integrated Chinese and Western Medicine Cancer Hospital of the University of Chinese Academy of Science (Zhejiang Cancer Hospital) Hangzhou China; ^18^ Cancer Center The First Hospital of Jilin University Changchun China; ^19^ Department of Radiation Oncology Sichuan Cancer Hospital and Institute, Sichuan Cancer Center, School of Medicine, University of Electronic Science and Technology of China Chengdu China; ^20^ Department of Gastrointestinal Surgery and Department of Clinical Nutrition Beijing Shijitan Hospital, Capital Medical University Beijing China; ^21^ Key Laboratory of Cancer FSMP for State Market Regulation Beijing China

**Keywords:** cancer, peridiagnosis, survival, weight change, weight gain, weight loss

## Abstract

**Background:**

Body weight and its changes have been associated with cancer outcomes. However, the associations of short‐term peridiagnosis weight dynamics in standardized, clinically operational time frames with cancer survival remain largely unknown. This study aimed to screen for and evaluate the optimal indicator of short‐term peridiagnosis weight dynamics to predict overall survival (OS) in patients with cancer.

**Methods:**

This multicentre cohort study prospectively collected data from 7460 patients pathologically diagnosed with cancer between 2013 and 2019. Body weight data were recorded 1 month before, at the time of and 1 month following diagnosis. By permuting different types (point value in kg, point height‐adjusted value in kg/m^2^, absolute change in kg or relative change in percentage) and time frames (prediagnosis, postdiagnosis or peridiagnosis), we generated 12 different weight‐related indicators and compared their prognostic performance using Harrell's C‐index, integrated discrimination improvement, continuous net reclassification improvement and time‐dependent C‐index. We analysed associations of peridiagnosis relative weight change (RWC) with OS using restricted cubic spine (RCS), Kaplan–Meier analysis and multivariable‐adjusted Cox regression models.

**Results:**

The study enrolled 5012 males and 2448 females, with a median age of 59 years. During a median follow‐up of 37 months, 1026 deaths occurred. Peridiagnosis (1 month before diagnosis to 1 month following diagnosis) RWC showed higher prognostic performance (Harrell's C‐index = 0.601, 95% confidence interval [CI] = [0.583, 0.619]) than other types of indicators including body mass index (BMI), absolute weight change, absolute BMI change, prediagnosis RWC and postdiagnosis RWC in the study population (all *P* < 0.05). Time‐dependent C‐index analysis also indicated that peridiagnosis RWC was optimal for predicting OS. The multivariable‐adjusted RCS analysis revealed an N‐shaped non‐linear association between peridiagnosis RWC and OS (*P*_*RWC*_ < 0.001, *P*_*non‐linear*_ < 0.001). Univariate survival analysis showed that the peridiagnosis RWC groups could represent distinct mortality risk stratifications (*P* < 0.001). Multivariable survival analysis showed that, compared with the maintenance group (weight change < 5%), the significant (gain >10%, hazard ratio [HR] = 0.530, 95% CI = [0.413, 0.680]) and moderate (gain 5–10%, HR = 0.588, 95% CI = [0.422, 0.819]) weight gain groups were both associated with improved OS. In contrast, the moderate (loss 5–10%, HR = 1.219, 95% CI = [1.029, 1.443]) and significant (loss >10%, HR = 1.280, 95% CI = [1.095, 1.497]) weight loss groups were both associated with poorer OS.

**Conclusions:**

The prognostic performance of peridiagnosis RWC is superior to other weight‐related indicators in patients with cancer. The findings underscore the importance of expanding the surveillance of body weight from at diagnosis to both past and future, and conducting it within clinically operational time frames, in order to identify and intervene with patients who are at risk of weight change‐related premature deaths.

## Introduction

The importance of body size on cancer prognosis is a topic of growing medical concerns worldwide.^1^, ^2^ Body mass index (BMI) is the most commonly used measure of body size, and its prognostic role in cancer has been extensively investigated.^3^, ^4^, ^5^, ^6^, ^7^ However, the results are still subject to debate: While high BMI is linked to poorer survival in breast cancer,^8^ it may improve survival in lung,^4^ gastric^5^ and colon cancers.^7^ The ‘obesity paradox’ in oncology populations also adds complexity,^3^, ^9^, ^10^ which may be explained by reasons such as methodologic limitations and heterogeneity in study design and population.^10^ BMI's limitation lies in capturing only a static body weight measure, while patients with cancer commonly experience drastic weight changes due to various factors.^11^, ^12^ Therefore, it is suggested that weight change over time may be more relevant than BMI alone in providing greater prognostic insights.^2^, ^3^, ^13^

Weight loss is prevalent in oncology practice^2^ and is a predominant diagnostic criterion for cancer cachexia^14^ and malnutrition.^15^ Previous studies showed that it is independently associated with worse survival in various cancers,^2^ such as oesophageal cancer,^16^ lung cancer^17^ and colorectal cancer, and the severity of weight loss is proportional to the death hazard.^18^ In contrast, postdiagnosis weight gain has been related to higher mortality in prostate cancer^13^ and head and neck cancer,^19^ but not in lung cancer.^17^

The association between weight changes and cancer mortality is complicated by factors such as the timing of data collection and the primary treatment received.^2^, ^3^, ^13^, ^16^, ^17^, ^18^, ^20^ As the accuracy of recalled weight decreases as a patient's age and/or the elapsed time increases,^21^ short‐term prediagnosis weight change may be more accurate than long‐term or early life weight data.^2^, ^13^, ^22^ Similarly, drastic body weight changes are likely to occur during anticancer treatment,^20^ making short‐term postdiagnosis weight changes more clinically relevant and easily modified^13^ for informing survivor recommendations. Additionally, some studies use unfixed time frames to calculate weight change, leading to uncertainty in clinical application.^2^, ^13^ Finally, the optimal use of multi‐point peridiagnosis weight data for cancer prognosis is still unclear.

Therefore, we sought to investigate the association of short‐term peridiagnosis weight dynamics in standardized time frames with cancer survival. We especially focused on the superiority of different weight‐related indicators for predicting survival. This work aimed to provide evidence to help develop clinically generalizable body weight surveillance algorithms to optimize patient prognosis in cancer care.

## Methods

### Study design and population

This was a multicentre observational cohort study with prospectively collected data. Patients were derived from a nationwide project, the Investigation on Nutrition Status and its Clinical Outcome of Common Cancers (INSCOC), which was registered online at http://www.chictr.org.cn/showproj.aspx?proj=31813 (ChiCTR1800020329). Detailed information about INSCOC has been described elsewhere,^23^ and the full inclusion and exclusion criteria are shown in *Table* *S1*. Based on these criteria, we included 7476 patients who were first diagnosed with nine types of cancer (lung, nasopharyngeal, colorectal, gastric, oesophageal, liver, pancreatic, biliary tract and gastric stromal cancers) and were hospitalized for anticancer treatment from January 2013 to April 2019. We only included patients for whom electronic peridiagnosis weight data were available. We further excluded 16 patients who died within the first 30 days after admission due to the study design. This left 7460 patients for the formal analysis (*Figure* *S1*). The study was approved by the Ethics Committees of all participating institutions, and written consent was obtained from all patients.

### Data collection and handling

Baseline data were obtained through in‐person interviews and physical examinations by project‐trained researchers within the first 48 h after patient admission: patient age, sex, smoking status (active tobacco smoker in the past 1 year, regardless of amount) and alcohol drinking (once a week or more frequent alcohol consumption in the past 1 year, regardless of type and amount), residency (urban vs. rural), food intake (normal vs. reduced, defined as intake below 50–75% of the normal requirement in the preceding week) and the Eastern Cooperative Oncology Group (ECOG) physical performance score.

Clinical characteristics recorded during hospitalization were retrospectively retrieved from electronic medical records after patient discharge. These included the pathology‐confirmed cancer type (also merged as gastrointestinal cancer and respiratory cancer in stratified analyses), clinical tumour stage, anticancer therapies (curative‐intent surgery, adjuvant chemotherapy and curative‐intent chemotherapy, encoded as binary variables) and nutritional intervention (parenteral and/or enteral).

### Assessment of body mass index and weight change

Body weight 1 month before (1 mon−), at the time of (baseline) and 1 month following diagnosis (1 mon+) were investigated. Past weight was reported by patients during the interview. Baseline body weight and height were measured using a scale with integrated height measurement in patients wearing light indoor clothing without shoes to the nearest 0.1 kg and 0.1 cm, respectively, within 48 h of the first admission. Weight 1 mon+ was either measured (if hospital stay ≥ 30 days) or reported by patients via follow‐up after discharge (if hospital stay < 30 days). When collecting patient‐reported weight information, the specific type/model of weight scale used by the patients was not considered. BMI was calculated as weight in kilograms divided by baseline height in metres squared (kg/m^2^), which was also grouped as underweight (<18.5), normal (18.5 to <24), overweight (24 to <28) or obese (≥28) according to the Chinese recommendations.^24^

Absolute weight change (AWC, kg), absolute BMI change (ABC, kg/m^2^) and relative weight change (RWC, %) were independently calculated for the following three time frames: prediagnosis (from 1 mon− to baseline), postdiagnosis (from baseline to 1 mon+) and peridiagnosis (from 1 mon− to 1 mon+), respectively. Accordingly, nine different weight change indicators were generated. AWC and ABC were calculated as follows: (updated weight in kg − previous weight in kg) and (updated BMI in kg/m^2^ − previous BMI in kg/m^2^), respectively. RWC was calculated as

$$\left(\frac{{\text{weight}}_{\text{updated}}-{\text{weight}}_{\text{previous}}}{{\text{weight}}_{\text{previous}}}\right)\times 100\%$$

The RWC was categorized as significant gain (>10%), moderate gain (5–10%), maintenance (<5%), moderate loss (5–10%) and significant loss (>10%), according to expert recommendations^3^ and an international consensus guideline.^15^

### Exposure, primary outcome and follow‐up

The weight‐related indicators (nine weight change indicators and BMI at three time points) were the exposure variables of the study. Patients were followed annually after admission via telephone or in‐person interviews to obtain their survival status. All‐cause mortality was the primary outcome, and the overall survival (OS) time was calculated as the time interval (months) between the first admission and the patient's date of death, the date of the last valid follow‐up or April 2020.

### Statistical analyses

Continuous data were presented as medians [25th percentile, 75th percentile] and compared using a nonparametric Wilcoxon's rank‐sum test. Categorical data were expressed as numbers (percentages) and compared using a *χ* ^2^ test. Harrell's C‐index,^25^ integrated discrimination improvement (IDI),^26^ continuous net reclassification improvement (cNRI)^27^ and time‐dependent C‐index estimation were calculated to assess and compare the prognostic performance of the various weight‐related indicators. The time‐dependent C‐index was calculated and visualized monthly within a 5‐year interval, following a 1000‐sample bootstrap cross‐validation to improve robustness.

A restricted cubic spline was used to flexibly analyse the potential dose‐dependent/non‐linear association of RWC with survival. The univariate association between RWC categories and survival was analysed using Kaplan–Meier curves following a log‐rank test. Cox proportional hazards regression models were used to calculate multivariable‐adjusted hazard ratios (HRs) and 95% confidence intervals (CIs). Incremental Cox regression models with increasing numbers of covariates were created. Model 1 was an unadjusted, crude model. Model 2 was adjusted for age at baseline and sex. Model 3 was adjusted for all variables in Model 2, plus smoking, drinking, residency, cancer type, clinical stage, surgery, adjuvant chemotherapy, curative chemotherapy, nutritional intervention, food intake and the ECOG. To mitigate the potential for bias as a result of reverse causation, sensitivity analyses were performed to test the robustness of the multivariate Cox regression models by excluding the patients who died within the first 3 months (Model 4), 6 months (Model 5) and 12 months (Model 6), respectively. Stratified analyses were performed in strata of patient age, sex, cancer type, clinical stage, BMI, surgery, adjuvant chemotherapy, curative chemotherapy, nutritional intervention and length of hospital stay to study effect modifications. Multiplicative interactions were tested by adjusting the cross‐product terms. Kaplan–Meier curves and the Schoenfeld individual test were used to visually and statistically estimate the proportional hazards assumption (Schoenfeld's test *P* > 0.05 indicates that the proportional hazards assumption is satisfied). The linearity assumption between covariates and outcome was confirmed by the Martingale residual plots. All tests were two‐sided, and *P* < 0.05 was regarded as statistically significant. The analyses were performed using R Version 3.6.3 (Foundation for Statistical Computing, Vienna, Austria).

## Results

### Baseline characteristics and weight‐related indicators

**Figure 1:**
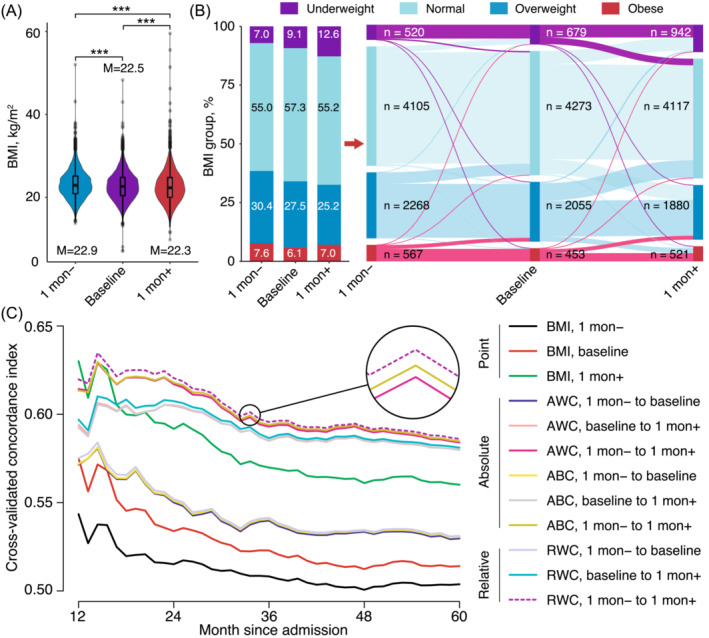
Weight‐related indicators and overall survival. 1 mon−, 1 month before cancer diagnosis; 1 mon+, 1 month after cancer diagnosis; ABC, absolute BMI change; AWC, absolute weight change; baseline, at diagnosis; BMI, body mass index; M, median; RWC, relative weight change. (A) Distribution of BMI at the 1 mon−, baseline and 1 mon+ time points. (B) Percentage, number and flow of the BMI categories at different time points. (C) Comparison of the time‐dependent C‐indices for different weight‐related indicators. All curves were corrected with 100 repetitions of 1000‐sample bootstrap cross‐validation.

A total of 7460 patients (5012 males and 2448 females), with a median age of 59.0 years, were included in the study. The most common tumour locations were the lung (29.0%), colorectum (25.5%), stomach (15.4%) and nasopharynx (15.2%). The majority of patients were at clinical stages III (36.6%) and II (33.0%). The median BMIs at 1 mon−, baseline and 1 mon+ were 22.9, 22.5 and 22.3 kg/m^2^, respectively (*Table* *1*). Overall, there was a significant trend of weight loss observed across the three time points (*Figure* *1* *A*). Changes in the proportions and numbers for the BMI category over the three time points are shown in *Figure* *1* *B*. Briefly, both weight gain and weight loss were observed at the group level.

**Table 1:** Baseline characteristics of the study population

Characteristics	Value
Patients	7460
Age, years	59.0 [51.0, 66.0]^a^
Sex, male	5012 (67.2)^b^
Smoking	3615 (48.5)
Alcohol drinking	1745 (23.4)
Residency, urban	4545 (60.9)
Cancer site	
Lung	2162 (29.0)
Colorectum	1903 (25.5)
Stomach	1148 (15.4)
Nasopharynx	1137 (15.2)
Oesophagus	704 (9.4)
Liver	283 (3.8)
Pancreas	64 (0.9)
Biliary tract	39 (0.5)
Gastric stroma	20 (0.3)
Cancer site merged, respiratory	3299 (44.2)
Clinical stage	
I	701 (9.4)
II	2462 (33.0)
III	2728 (36.6)
IV	1569 (21.0)
Curative‐intent surgery, yes	3022 (40.5)
Adjuvant chemotherapy, yes	1620 (21.7)
Curative‐intent chemotherapy, yes	1123 (15.1)
Nutritional intervention, yes	2305 (30.9)
Food intake, normal	22.9 [20.8, 25.1]
ECOG physical performance score (%)	
0	5374 (72.0)
1	1747 (23.4)
2	196 (2.6)
3	107 (1.4)
4	36 (0.5)
Body mass index, kg/m^2^, 1 month before baseline	22.9 [20.8, 25.1]
Body mass index, kg/m^2^, baseline	22.5 [20.4, 24.8]
Body mass index, kg/m^2^, 1 month after baseline	22.3 [20.0, 24.8]

Abbreviation: ECOG, the Eastern Cooperative Oncology Group.

^a^ Median (interquartile range), all such values.

^b^ Number (percentage), all such values.

### Prognostic performance of weight‐related indicators

There were 1026 deaths among 7460 patients during a median follow‐up period of 37 months. The results of Harrell's C‐index, IDI and cNRI for the 12 weight‐related indicators are shown in *Table* *2*. Peridiagnosis RWC demonstrated the highest C‐index (0.601, 95% CI = [0.583, 0.619]) for predicting OS among all investigated indicators (all *P* < 0.05). The IDI and cNRI results were consistent, showing that RWC had statistically significant discrimination improvement compared with the other indicators (all *P* < 0.05) except for BMI 1 mon+ (*P* for IDI = 0.376, *P* for cNRI = 0.317).

**Table 2:** Prognostic performance of different weight‐related indicators

Index	Harrell's C‐index (95% CI)	*P*	IDI (95% CI)	*P*	cNRI (95% CI)	*P*
Point BMI value, kg/m^2^						
1 mon−	0.518 (0.498, 0.538)	<0.001	−0.010 (−0.014, −0.006)	<0.001	−0.112 (−0.145, −0.070)	<0.001
Baseline	0.530 (0.510, 0.550)	<0.001	−0.009 (−0.014, −0.005)	<0.001	−0.115 (−0.143, −0.068)	<0.001
1 mon+	0.578 (0.558, 0.598)	0.020	−0.002 (−0.005, 0.003)	0.376	−0.021 (−0.082, 0.021)	0.317
Absolute weight change, kg						
1 mon− to baseline	0.543 (0.525, 0.561)	<0.001	−0.009 (−0.013, −0.005)	<0.001	−0.112 (−0.147, −0.052)	<0.001
Baseline to 1 mon+	0.588 (0.572, 0.604)	0.005	−0.004 (−0.007, −0.002)	<0.001	−0.108 (−0.146, −0.050)	<0.001
1 mon− to 1 mon+	0.598 (0.580, 0.616)	<0.001	−0.002 (−0.003, −0.001)	<0.001	−0.115 (−0.158, −0.057)	<0.001
Absolute BMI change, kg/m^2^						
1 mon− to baseline	0.544 (0.526, 0.562)	<0.001	−0.008 (−0.013, −0.005)	<0.001	−0.110 (−0.149, −0.051)	<0.001
Baseline to 1 mon+	0.588 (0.572, 0.604)	0.006	−0.004 (−0.007, −0.002)	<0.001	−0.107 (−0.142, −0.060)	<0.001
1 mon− to 1 mon+	0.599 (0.581, 0.617)	0.014	−0.002 (−0.003, −0.001)	<0.001	−0.112 (−0.151, −0.058)	<0.001
Relative weight change, %						
1 mon− to baseline	0.545 (0.527, 0.563)	<0.001	−0.008 (−0.013, −0.004)	<0.001	−0.095 (−0.146, −0.042)	<0.001
Baseline to 1 mon+	0.590 (0.574, 0.606)	0.015	−0.003 (−0.005, −0.001)	0.020	−0.077 (−0.117, −0.022)	<0.001
1 mon− to 1 mon+	0.601 (0.583, 0.619)	Reference	Reference	—	Reference	—

Abbreviations: 1 mon−, 1 month before diagnosis; 1 mon+, 1 month following diagnosis; baseline, at the time of diagnosis; BMI, body mass index; CI, confidence interval; cNRI, continuous net reclassification improvement; IDI, integrated discrimination improvement.

The values of the time‐dependent C‐index analysis are shown in *Figure* *1* *C*. Overall, peridiagnosis RWC exhibited optimal prognostic performance among all investigated indicators. Regarding point values, BMI 1 mon+ demonstrated higher prognostic performance compared with baseline BMI and BMI 1 mon−. Peridiagnosis weight change showed superior prognostic performance over prediagnosis and postdiagnosis weight changes, regardless of the indicator type (AWC, ABC or RWC). Additionally, RWC showed better prognostic performance than AWC and ABC across different time frames. These visually observed results were statistically confirmed by additional in‐group comparisons for Harrell's C‐index (all *P* < 0.05; *Table* *S2*). Interestingly, adjusting for height statistically improved the prognostic performance of the weight data 1 mon− (*P* = 0.049), but not for the other two time points. Taken together, the peridiagnosis RWC from 1 mon− to 1 mon+ was deemed optimal and selected for future analysis.

### Relation of weight change with overall survival

**Figure 2:**
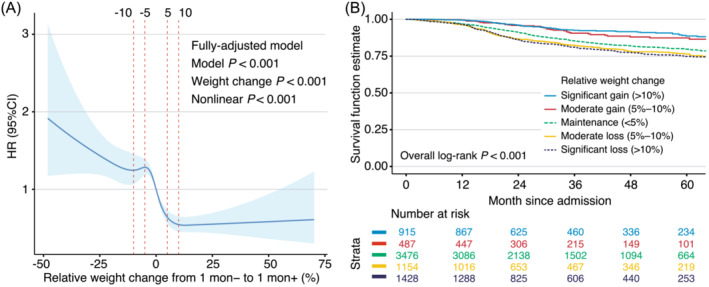
Peridiagnosis weight change and survival. (A) Dose–response association between peridiagnosis relative weight change and overall survival. Associations were examined by multivariable Cox regression models based on restricted cubic splines. The blue solid line represents estimates of hazard ratios (HRs), and the blue ribbon represents 95% confidence intervals (CIs). Risk estimates were adjusted for baseline age, sex, smoking, drinking, residency, cancer type, clinical stage, surgery, adjuvant chemotherapy, curative chemotherapy, nutritional intervention, food intake and the Eastern Cooperative Oncology Group physical performance score. (B) Kaplan–Meier analysis on the association between categories of peridiagnosis relative weight change and overall survival.

Using zero as the reference, a fully adjusted restricted cubic spline analysis revealed that peridiagnosis RWC was independently associated with OS (*P* < 0.001) in a non‐linear manner (*P*_*non‐linear*_ < 0.001; *Figure* *2* *A*). After stratification, there were 915 (12.3%), 487 (6.5%), 3476 (46.6%), 1154 (15.5%) and 1428 (19.1%) patients in the significant weight gain, moderate weight gain, maintenance, moderate weight loss and significant weight loss groups, respectively. The baseline characteristics of the study population, stratified by the peridiagnosis RWC category, are shown in *Table* *3*. Kaplan–Meier curves showed that the RWC category was associated with OS, demonstrating distinct risk stratifications (log‐rank *P* < 0.001; *Figure* *2* *B*).

**Table 3:** Baseline characteristics stratified by peridiagnosis weight change category

Characteristics	Relative weight change from 1 month before baseline to 1 month after baseline					*P* value^a^
	Significant gain (>10%)	Moderate gain (5–10%)	Maintenance (<5%)	Moderate loss (5–10%)	Significant loss (>10%)	
Patients	915 (12.3)	487 (6.5)	3476 (46.6)	1154 (15.5)	1428 (19.1)	—
Age, years	57.0 [48.5, 64.0]^b^	56.0 [50.0, 64.0]	59.0 [50.0, 66.0]	60.0 [52.0, 66.0]	60.0 [52.0, 66.0]	<0.001
Sex, male	565 (61.7)^c^	329 (67.6)	2321 (66.8)	767 (66.5)	1030 (72.1)	<0.001
Smoking, ever smoker	463 (50.6)	242 (49.7)	1631 (46.9)	563 (48.8)	716 (50.1)	0.140
Alcohol drinking, regular	209 (22.8)	133 (27.3)	745 (21.4)	282 (24.4)	376 (26.3)	0.001
Residency, urban	549 (60.0)	314 (64.5)	2077 (59.8)	718 (62.2)	887 (62.1)	0.160
Cancer site						<0.001
Lung	334 (36.5)	169 (34.7)	1088 (31.3)	273 (23.7)	298 (20.9)	
Colorectum	320 (35.0)	150 (30.8)	933 (26.8)	283 (24.5)	217 (15.2)	
Stomach	80 (8.7)	46 (9.4)	413 (11.9)	214 (18.5)	395 (27.7)	
Nasopharynx	89 (9.7)	62 (12.7)	582 (16.7)	195 (16.9)	209 (14.6)	
Oesophagus	51 (5.6)	29 (6.0)	252 (7.2)	126 (10.9)	246 (17.2)	
Liver	25 (2.7)	23 (4.7)	166 (4.8)	45 (3.9)	24 (1.7)	
Pancreas	7 (0.8)	3 (0.6)	20 (0.6)	11 (1.0)	23 (1.6)	
Biliary tract	5 (0.5)	2 (0.4)	14 (0.4)	6 (0.5)	12 (0.8)	
Gastric stroma	4 (0.4)	3 (0.6)	8 (0.2)	1 (0.1)	4 (0.3)	
Cancer site merged, respiratory	423 (46.2)	231 (47.4)	1670 (48.0)	468 (40.6)	507 (35.5)	<0.001
Clinical stage						0.002
I	71 (7.8)	48 (9.9)	365 (10.5)	93 (8.1)	124 (8.7)	
II	338 (36.9)	178 (36.6)	1141 (32.8)	355 (30.8)	450 (31.5)	
III	328 (35.8)	171 (35.1)	1217 (35.0)	448 (38.8)	564 (39.5)	
IV	178 (19.5)	90 (18.5)	753 (21.7)	258 (22.4)	290 (20.3)	
Curative surgery, yes	467 (51.0)	247 (50.7)	1387 (39.9)	440 (38.1)	481 (33.7)	<0.001
Adjuvant chemotherapy, yes	268 (29.3)	141 (29.0)	802 (23.1)	201 (17.4)	208 (14.6)	<0.001
Curative chemotherapy, yes	156 (17.0)	87 (17.9)	527 (15.2)	173 (15.0)	180 (12.6)	0.013
Nutritional intervention, yes	261 (28.5)	141 (29.0)	891 (25.6)	411 (35.6)	601 (42.1)	<0.001
Food intake, normal	594 (64.9)	311 (63.9)	2287 (65.8)	673 (58.3)	754 (52.8)	<0.001
ECOG score (%)						<0.001
0	670 (73.2)	359 (73.7)	2575 (74.1)	785 (68.0)	985 (69.0)	
1	200 (21.9)	105 (21.6)	763 (22.0)	311 (26.9)	368 (25.8)	
2	23 (2.5)	18 (3.7)	85 (2.4)	31 (2.7)	39 (2.7)	
3	22 (2.4)	4 (0.8)	35 (1.0)	21 (1.8)	25 (1.8)	
4	0 (0.0)	1 (0.2)	18 (0.5)	6 (0.5)	11 (0.8)	
BMI, kg/m^2^, 1 mon−	21.1 [19.2, 23.4]	22.6 [20.3, 24.8]	23.0 [20.9, 25.0]	23.1 [21.2, 25.4]	23.9 [21.9, 26.0]	<0.001
BMI, kg/m^2^, baseline	21.0 [19.2, 23.4]	22.5 [20.2, 24.7]	22.7 [20.6, 24.8]	22.6 [20.4, 24.8]	23.0 [20.8, 25.2]	<0.001
BMI, kg/m^2^, 1 mon+	24.5 [22.3, 27.2]	24.2 [21.5, 26.5]	22.9 [20.8, 25.0]	21.5 [19.5, 23.6]	19.6 [17.7, 21.6]	<0.001

Abbreviations: 1 mon−, 1 month before diagnosis; 1 mon+, 1 month following diagnosis; baseline, at the time of diagnosis; BMI, body mass index; ECOG, the Eastern Cooperative Oncology Group.

^a^ Kruskal–Wallis rank‐sum test and *χ* ^2^ test were used to calculate *P* values for categorical variables and continuous variables, respectively.

^b^ Median (interquartile range), all such values.

^c^ Number (percentage), all such values.

### Multivariable‐adjusted Cox regression models

The results of the multivariable Cox proportional hazards model analyses for the RWC categories and OS are shown in *Table* *4*. Compared with patients who maintained their weight, those with significant weight gain (HR = 0.530, 95% CI = [0.413, 0.680]) and moderate weight gain (HR = 0.588, 95% CI = [0.422, 0.819]) were independently associated with a reduced death hazard in the fully adjusted model (Model 3). Conversely, moderate weight loss (HR = 1.219, 95% CI = [1.029, 1.443]) and significant weight loss (HR = 1.280, 95% CI = [1.095, 1.497]) were both associated with an increased death hazard. Sensitivity analyses showed that these associations remained significant after excluding patients who died within the first 3 months (Model 4), 6 months (Model 5) and 12 months (Model 6) after enrolment.

**Table 4:** Hazard ratios and 95% confidence intervals of peridiagnosis weight change with overall survival

All‐cause mortality	Relative weight change from 1 month before baseline to 1 month after baseline				
	Significant gain (>10%)	Moderate gain (5–10%)	Maintenance (<5%)	Moderate loss (5–10%)	Significant loss (>10%)
All patients					
Events/no.	72/915	38/487	472/3476	192/1154	252/1428
Model 1^a^	0.508 (0.397, 0.651)	0.551 (0.396, 0.766)	1 [reference]	1.282 (1.084, 1.516)	1.331 (1.142, 1.551)
Model 2^b^	0.533 (0.415, 0.683)	0.564 (0.405, 0.785)	1 [reference]	1.251 (1.058, 1.480)	1.286 (1.103, 1.498)
Model 3^c^	0.530 (0.413, 0.680)	0.588 (0.422, 0.819)	1 [reference]	1.219 (1.029, 1.443)	1.280 (1.095, 1.497)
Sensitivity analysis 1					
Events/no.	72/912	38/485	454/3357	186/1119	245/1411
Model 4^d^	0.549 (0.427, 0.704)	0.613 (0.440, 0.854)	1 [reference]	1.231 (1.036, 1.462)	1.300 (1.109, 1.524)
Sensitivity analysis 2					
Events/no.	72/911	37/481	428/3300	179/1102	230/1382
Model 5^e^	0.581 (0.452, 0.747)	0.632 (0.451, 0.885)	1 [reference]	1.270 (1.065, 1.515)	1.310 (1.112, 1.543)
Sensitivity analysis 3					
Events/no.	68/867	36/447	371/3086	151/1016	205/1288
Model 6^f^	0.627 (0.483, 0.813)	0.705 (0.500, 0.994)	1 [reference]	1.251 (1.034, 1.514)	1.375 (1.155, 1.637)

^a^ Model 1 was an unadjusted, crude model.

^b^ Model 2 was adjusted for baseline age (continuous) and sex (reference = female).

^c^ Model 3 was adjusted for baseline age (continuous), sex (reference = female), smoking (reference = no), drinking (reference = no), residency (reference = rural), cancer type (reference = gastrointestinal cancer), clinical stage (reference = I), surgery (reference = no), adjuvant chemotherapy (reference = no), curative chemotherapy (reference = no), any parenteral and/or enteral nutritional intervention (reference = no), food intake (reference = reduced) and the Eastern Cooperative Oncology Group (ECOG) score (reference = 0).

^d^ Model 4 was adjusted for all variables in Model 3, but excluded those patients who died within the first 3 months after enrolment.

^e^ Model 5 was adjusted for all variables in Model 3, but excluded those patients who died within the first 6 months after enrolment.

^f^ Model 6 was adjusted for all variables in Model 3, but excluded those patients who died within the first 12 months after enrolment.

### Stratified analysis

The fully adjusted models were repeated in different variable subgroups including age (<60 vs. ≥60), sex (female vs. male), cancer type (respiratory cancer vs. gastrointestinal cancer), clinical stage (I–II vs. III–IV), BMI (<24 vs. ≥24 kg/m^2^), surgery (no vs. yes), adjuvant chemotherapy (no vs. yes), curative chemotherapy (no vs. yes), nutritional intervention (no vs. yes) and length of hospital stay (<30 vs. ≥30 days) to examine effect modifications (*Table* *S3*). For the weight gain groups, the protective effect of significant weight gain was sustained in all subgroups except for patients who received nutritional intervention and those with longer hospital stays. Additionally, the protective effect of moderate weight gain persisted in patients with older age, female sex, gastrointestinal cancer, higher tumour stages, lower BMI, surgery (yes and no), adjuvant chemotherapy (yes and no), curative chemotherapy (no), nutritional intervention (no) and a shorter length of hospital stay. However, this effect was attenuated in other subgroups. For the moderate weight loss group, the positive association with mortality was sustained in patients with younger age, respiratory cancer, a higher BMI, surgery (no), adjuvant chemotherapy (no), curative chemotherapy (no), nutritional intervention (no) and a shorter length of hospital stay. Additionally, the positive association of significant weight loss with mortality was only sustained in patients with younger age, male sex, respiratory cancer, higher tumour stages, lower BMI, surgery (no), adjuvant chemotherapy (no), curative chemotherapy (yes and no), nutritional intervention (no) and a shorter length of hospital stay.

## Discussion

In this report, we address several knowledge gaps about short‐term peridiagnosis weight dynamics and OS in patients with common cancers. We used data from a multicentre, nationally representative project with standardized assessment and follow‐up. Our study demonstrates that peridiagnosis RWC from 1 mon− to 1 mon+ was the optimal indicator to predict OS and also revealed that peridiagnosis weight gain and weight loss were monotonically associated with improved and reduced OS, respectively. The findings underscore the importance of expanding surveillance of body weight from baseline to both past and future directions within clinically operational time frames to provide significant prognostic information. While clinicians' instinct may be to prioritize those with weight loss,^2^, ^14^, ^15^ our results warrant attention to peridiagnosis weight gain during the cancer journey, leading us to suggest establishing a system of cancer‐associated weight gain to inform novel intervention strategies.

BMI and weight change are important indicators of the presence, severity and progress of many diseases,^28^ including nutritional disorders such as malnutrition^15^ and cachexia.^14^ In general, weight changes (gain or loss) in early adulthood have been related to all‐cause and cancer‐specific mortality.^22^, ^29^ However, instead of being measured and retrospectively retrieved in prospectively designed research settings, weight loss was ‘naturally’ calculated based on patient‐reported previous weight in newly diagnosed cancer in clinical settings.^30^, ^31^ Recollection of weight data after months is reported to be less reliable.^28^ A previous study conducted in patients with pancreatic cancer suggests that postdiagnosis weight loss over 60 days did not predict poor prognosis.^32^ Because we also employed a short time frame, these results support the superiority of our timing strategy in this study. Future studies need to explore the prognostic significance of more weight change time frames that vary in length and timing.

The associations between weight loss, weight gain and survival in cancer may be attributed to different underlying mechanisms.^33^ Our results are consistent with previous studies indicating a negative association of weight loss with cancer survival.^14^, ^34^ However, our study is unique in examining peridiagnosis weight gain and OS. A previous study showed that patients with prediagnosis or postdiagnosis weight gain generally have better outcomes compared with those who experienced weight loss.^34^ Additionally, early intervention to maintain body weight and nutritional status may improve the efficacy of immune checkpoint inhibitors in patients with gastric cancer.^35^ Our observations partially align with this report, showing that peridiagnosis weight gain is protective for survival. However, weight gain, especially significant weight gain,^34^ can also be a risk factor for OS.^33^ Elucidating the true nature of weight gain during the onset and development of cancer is difficult.^34^ On one hand, obesity might mask the existence of sarcopenia (sarcopenic obesity).^36^ On the other hand, weight gain may also represent a milder degree of disease associated with adequate food intake and the absence of cachexia or reversible cancer cachexia. Therefore, weight gain as a factor may have a variable impact on survival. In this study, patients with significant and moderate weight gain showed higher rates of normal food intake at diagnosis compared with those with moderate and significant weight loss (*Table* *3*). Additionally, our previous studies conducted on the same INSCOC cohort also found a favourable effect of fat mass storage, both subcutaneous^37^ and whole body fats,^38^ on cancer survival. Future studies need to replicate our results with a greater variety of ethnic groups.

This study had several strengths, including a relatively large and nationally representative sample, standardized assessment and follow‐up, weight data at multiple time points and a detailed analysis of weight change. Uniquely, the study compared different weight‐related indicators in parallel over standardized time frames. These findings might provide useful information to help select clinically operational weight surveillance algorithms, such as timing, length of observation and type of weight change indicator. Another strength of our research is that we adjusted for a large number of potential confounders, taking into account socio‐economic status, lifestyle factors, cancer type and severity, anticancer therapies, nutritional interventions, diet quality and physical activity. Additionally, the cut‐offs used to categorize RWC were widely employed by previous studies and international guidelines,^3^, ^14^, ^15^, ^33^, ^39^ supporting their generalizability in future clinical applications.

Our study also has several limitations that must be noted: First, all the prediagnosis weight data and part of the postdiagnosis weight data were recalled or self‐reported by patients, which might introduce misclassification bias. However, we used 1‐month short time frames and only included data from those patients who could provide their past weight in the study, partially reducing the possibility. According to our database, 4.5% of patients were unable to report their weight 1 month ago, and 47.4% were unable to report their weight 6 months ago (data not shown). This at least partially supports the effectiveness of our efforts in controlling the risk of recall bias. Nevertheless, future studies with a prospective design and measured weight change information are needed to replicate our results. Second, as obesity can mask sarcopenia while related variables were not collected for this study, future studies incorporating skeletal muscle measures and other body composition parameters should provide greater insights. Third, we observed some effect modifications in the stratified analyses. However, the associations between various weight change groups and survival were only attenuated in effect without completely changing direction. Thus, the small numbers/events in these groups may have contributed to this variability. Future studies employing a larger sample size are needed to confirm our findings, especially in more specific initial BMI categories. Fourth, we only collected the covariates at baseline, so we could not adjust time‐varying covariates to capture changes in possible confounders over time. Fifth, due to the limited scope of the original INSCOC project, data on diet structure, exercise status and food intake following hospitalization were unavailable and therefore not adjusted in the multivariable survival analysis. While the adjusted baseline food intake and ECOG score may partially reflect information on these dimensions, it is important for future studies to determine whether adjusting these variables would alter the observed associations. Additionally, the time dimension of the received anticancer therapy was not documented in this study due to its limited scope (e.g., time to surgery). Subsequent studies should explore the potential effect modification induced by this factor. Finally, the results should be interpreted with caution when applied to other cancer types and ethnic groups. It is essential to validate the observed associations in a more diverse range of patient populations to enhance the reliability of the study findings.

Our study found that peridiagnosis RWC (1 mon− to 1 mon+) showed better prognostic performance to predict OS than BMI, absolute weight/BMI changes, prediagnosis RWC and postdiagnosis RWC. Peridiagnosis weight gain and weight loss were monotonically associated with improved and reduced OS, respectively. Future studies are needed to unravel the mechanisms underlying the association of short‐term peridiagnosis weight dynamics, particularly the associations of changes in body composition, with OS and other outcomes in patients with cancer.

## Conflict of interest

Liangyu Yin, Ling Zhang, Long Li, Ming Liu, Jin Zheng, Aiguo Xu, Quanjun Lyu, Yongdong Feng, Zengqing Guo, Hu Ma, Jipeng Li, Zhikang Chen, Hui Wang, Zengning Li, Chunling Zhou, Xi Gao, Min Weng, Qinghua Yao, Wei Li, Tao Li, Hanping Shi and Hongxia Xu declare that they have no conflict of interest.

## Supporting information


**Table S1.** Inclusion and exclusion criteria for the Investigation on Nutrition Status and its Clinical Outcome of Common Cancers (INSCOC) project
**Table S2.** Additional comparison for the Harrell's C‐indices of different weight‐related measures
**Table S3.** Stratified analyses on the association of peridiagnosis weight change with all‐cause mortality
**Figure S1.** A flowchart of the patient inclusion
