# Supplementary material for: Exploring the optimal indicator of short‐term peridiagnosis weight dynamics to predict cancer survival: A multicentre cohort study
Source: J Cachexia Sarcopenia Muscle. 2024 Apr 21;15(3):1177–86. doi: 10.1002/jcsm.13467 (PMC11154758; doi:10.1002/jcsm.13467)
Supplement: Supplementary file 1 — Table S1. Inclusion and exclusion criteria for the Investigation on Nutrition Status and its Clinical Outcome of Common Cancers (INSCOC) project Table S2. Additional comparison for the Harrell's C‐indices of different weight‐related measures Table S3. Stratified analyses on the association of peridiagnosis weight change with all‐cause mortality Figure S1. A flowchart of the patient inclusion [file JCSM-15-1177-s001.pdf]

Title: Exploring the optimal indicator of short-term peridiagnosis weight dynamics to predict cancer survival: A multicenter cohort study

Authors: Liangyu Yin, Ling Zhang, Long Li, Ming Liu, Jin Zheng, Aiguo Xu, Quanjun Lyu, Yongdong Feng, Zengqing Guo, Hu Ma, Jipeng Li, Zhikang Chen, Hui Wang, Zengning Li, Chunling Zhou, Xi Gao, Min Weng, Qinghua Yao, Wei Li, Tao Li, Hanping Shi and Hongxia Xu

Supplementary files

Number	Item
1	**Table S1.** Inclusion and exclusion criteria for the Investigation on Nutrition Status and its Clinical Outcome of Common Cancers (INSCOC) project
2	**Table S2.** Additional comparison for the Harrell's C-indices of different weight-related measures
3	**Table S3.** Stratified analyses on the association of peridiagnosis weight change with all-cause mortality
4	**Figure S1.** A flowchart of the patient inclusion

Table S1. Inclusion and exclusion criteria for the INSCOC project

Inclusion criteria	Exclusion criteria
1) Age at least 18 years;<br>2) With length of hospital stay longer than 48 hours;<br>3) Diagnosed with one of the following 18 types of locally or metastatic malignant tumors: lung cancer, gastric cancer, liver cancer, colorectal cancer, breast cancer, esophageal cancer, cervical cancer, endometrial cancer, nasopharyngeal carcinoma, malignant lymphoma, leukemia, pancreatic cancer, ovarian cancer, prostate cancer, bladder cancer, brain tumors, biliary tract malignant tumors and gastrointestinal stromal tumors	1) With organ transplantation;<br>2) Pregnant woman;<br>3) Diagnosed with HIV infection or AIDS;<br>4) Admitted to the ICU at the beginning of recruitment;<br>5) If patients were hospitalized more than two times during the investigation, only the data from the first survey were included

Abbreviations: INSCOC, Investigation on Nutrition Status and its Clinical Outcome of Common Cancers (chictr.org.cn: ChiCTR1800020329); HIV, human immunodeficiency virus; AIDS, acquired immunodeficiency syndrome; ICU, intensive care unit.

Table S2. Additional comparison for the Harrell's C-indices of weight-related indicators

Index	Harrell's C-index (95%CI)	*P*
Point BMI data, kg/m^2		
One mon−	0.518 (0.498, 0.538)	<0.001
Baseline	0.530 (0.510, 0.550)	<0.001
One mon+	0.578 (0.558, 0.598)	Reference
Absolute weight change, kg		
One mon− to baseline	0.543 (0.525, 0.561)	<0.001
Baseline to one mon+	0.588 (0.572, 0.604)	0.028
One mon− to one mon+	0.598 (0.580, 0.616)	Reference
Absolute BMI change		
One mon− to baseline	0.544 (0.526, 0.562)	<0.001
Baseline to one mon+	0.588 (0.572, 0.604)	0.019
One mon− to one mon+	0.599 (0.581, 0.617)	Reference
Relative weight change		
One mon− to baseline	0.545 (0.527, 0.563)	<0.001
Baseline to one mon+	0.590 (0.574, 0.606)	0.015
One mon− to one mon+	0.601 (0.583, 0.619)	Reference
One mon− to baseline, type 1		
Absolute weight change, kg	0.543 (0.525, 0.561)	0.005
Absolute BMI change, kg/m^2	0.544 (0.526, 0.562)	0.042
Relative weight change, %	0.545 (0.527, 0.563)	Reference
Baseline to one mon+, type 1		
Absolute weight change, kg	0.588 (0.572, 0.604)	0.007
Absolute BMI change, kg/m^2	0.588 (0.572, 0.604)	0.049
Relative weight change, %	0.590 (0.574, 0.606)	Reference
One mon− to one mon+, type 1		
Absolute weight change, kg	0.598 (0.580, 0.616)	<0.001
Absolute BMI change, kg/m^2	0.599 (0.581, 0.617)	0.013
Relative weight change, %	0.601 (0.583, 0.619)	Reference
One mon− to baseline, type 2		
Absolute weight change, kg	0.543 (0.525, 0.561)	0.049
Absolute BMI change, kg/m^2	0.544 (0.526, 0.562)	Reference
Baseline to one mon+, type 2		
Absolute weight change, kg	0.588 (0.572, 0.604)	0.937
Absolute BMI change, kg/m^2	0.588 (0.572, 0.604)	Reference
One mon− to one mon+, type 2		
Absolute weight change, kg	0.598 (0.580, 0.616)	0.303
Absolute BMI change, kg/m^2	0.599 (0.581, 0.617)	Reference

Abbreviations: CI, confidence interval; BMI, body mass index; 1 mon−, one month before diagnosis; baseline, at the time of diagnosis; 1 mon+, 1 month following diagnosis.

Table S3. Stratified analyses on the association of peridiagnosis weight change with overall survival

Models[1]	Relative weight change from one month before baseline to one month after baseline				
	Significant gain (>10%)	Moderate gain (5%–10%)	Maintenance (<5%)	Moderate loss (5%–10%)	Significant loss (>10%)
All patients, events/no.	72/915	38/487	472/3476	192/1154	252/1428
	0.530 (0.413, 0.680)	0.588 (0.422, 0.819)	1 [Reference]	1.219 (1.029, 1.443)	1.280 (1.095, 1.497)
Age < 60, events/no.	33/530	21/280	198/1841	81/570	112/703
	0.497 (0.343, 0.721)	0.737 (0.469, 1.158)	1 [Reference]	1.326 (1.021, 1.721)	1.494 (1.178, 1.894)
Age ≥ 60, events/no.	39/385	17/207	274/1635	111/584	140/725
	0.571 (0.407, 0.801)	0.465 (0.284, 0.760)	1 [Reference]	1.103 (0.882, 1.379)	1.063 (0.863, 1.310)
Women, events/no.	30/350	7/158	144/1155	57/387	55/398
	0.617 (0.415, 0.919)	0.330 (0.154, 0.706)	1 [Reference]	1.237 (0.908, 1.684)	0.958 (0.697, 1.317)
Men, events/no.	42/565	31/329	328/2321	135/767	197/1030
	0.475 (0.344, 0.656)	0.692 (0.478, 1.003)	1 [Reference]	1.214 (0.991, 1.487)	1.401 (1.169, 1.679)
Respiratory cancer, events/no.	36/423	18/231	187/1670	78/468	97/507
	0.678 (0.472, 0.972)	0.699 (0.429, 1.137)	1 [Reference]	1.430 (1.093, 1.872)	1.807 (1.408, 2.320)
Gastrointestinal cancer, events/no.	36/492	20/256	285/1806	114/686	155/921
	0.456 (0.321, 0.646)	0.512 (0.325, 0.807)	1 [Reference]	1.092 (0.877, 1.360)	1.039 (0.851, 1.269)
Clinical stage I-II, events/no.	20/409	10/226	125/1506	51/448	65/574
	0.548 (0.340, 0.882)	0.535 (0.280, 1.022)	1 [Reference]	1.385 (0.995, 1.927)	1.225 (0.897, 1.672)
Clinical stage III-IV, events/no.	52/506	28/261	347/1970	141/706	187/854
	0.512 (0.382, 0.687)	0.588 (0.399, 0.865)	1 [Reference]	1.162 (0.954, 1.416)	1.272 (1.061, 1.527)
BMI, 1 mon−, <24 kg/m^2, events/no.	63/737	29/321	297/2153	115/688	139/726
	0.544 (0.414, 0.716)	0.665 (0.453, 0.975)	1 [Reference]	1.229 (0.988, 1.529)	1.398 (1.137, 1.719)
BMI, 1 mon−, ≥24 kg/m^2, events/no.	9/178	9/166	175/1323	77/466	113/702
	0.410 (0.210, 0.803)	0.389 (0.199, 0.761)	1 [Reference]	1.333 (1.017, 1.747)	1.274 (0.999, 1.624)
Surgery, no, events/no.	51/448	22/240	309/2089	133/714	179/947
	0.620 (0.459, 0.836)	0.548 (0.354, 0.846)	1 [Reference]	1.358 (1.105, 1.668)	1.392 (1.153, 1.681)
Surgery, yes, events/no.	21/467	16/247	163/1387	59/440	73/481
	0.369 (0.233, 0.583)	0.566 (0.338, 0.948)	1 [Reference]	1.121 (0.830, 1.514)	1.190 (0.897, 1.579)
Adjuvant chemotherapy, no, events/no.	63/647	29/346	356/2674	167/953	225/1220
	0.641 (0.489, 0.840)	0.587 (0.402, 0.858)	1 [Reference]	1.385 (1.151, 1.668)	1.463 (1.233, 1.737)
Adjuvant chemotherapy, yes, events/no.	9/268	9/141	116/802	25/201	27/208
	0.223 (0.112, 0.441)	0.488 (0.247, 0.966)	1 [Reference]	0.996 (0.643, 1.544)	0.749 (0.486, 1.154)
Curative chemotherapy, no, events/no.	58/759	27/400	367/2949	149/981	202/1248
	0.579 (0.438, 0.765)	0.562 (0.380, 0.831)	1 [Reference]	1.273 (1.050, 1.542)	1.260 (1.056, 1.503)
Curative chemotherapy, yes, events/no.	14/156	11/87	105/527	43/173	50/180

	0.419 (0.235, 0.745)	0.544 (0.291, 1.018)	1 [Reference]	1.277 (0.885, 1.841)	1.556 (1.104, 2.193)
Nutritional intervention, no, events/no.	54/654	30/346	383/2585	147/743	161/827
	0.509 (0.382, 0.678)	0.552 (0.381, 0.802)	1 [Reference]	1.374 (1.134, 1.663)	1.314 (1.090, 1.583)
Nutritional intervention, yes, events/no.	18/261	8/141	89/891	45/411	91/601
	0.646 (0.387, 1.078)	0.538 (0.260, 1.112)	1 [Reference]	1.029 (0.717, 1.478)	1.308 (0.972, 1.760)
Length of hospital stay, <30d, events/no.	61/835	36/457	438/3185	182/1040	219/1245
	0.473 (0.362-0.620)	0.563 (0.400-0.791)	1 [Reference]	1.247 (1.048-1.484)	1.200 (1.018-1.416)
Length of hospital stay, ≥30d, events/no.	11/80	2/30	34/291	10/114	33/183
	1.058 (0.516-2.168)	0.454 (0.107-1.924)	1 [Reference]	0.875 (0.428-1.790)	1.343 (0.819-2.201)

Abbreviation: BMI, body mass index; 1 mon−, 1 month before baseline.

[1]Models were adjusted for baseline age (continuous), sex (reference=female), smoking (reference=no), drinking (reference=no), residency (reference=rural), cancer type (reference=gastrointestinal cancer), clinical stage (reference=I), surgery (reference=no), adjuvant chemotherapy (reference=no), curative chemotherapy (reference=no), any parenteral and/or enteral nutritional intervention (reference=no), food intake (reference=reduced) and the Eastern Cooperative Oncology Group (ECOG) score (reference=0).

Figure S1

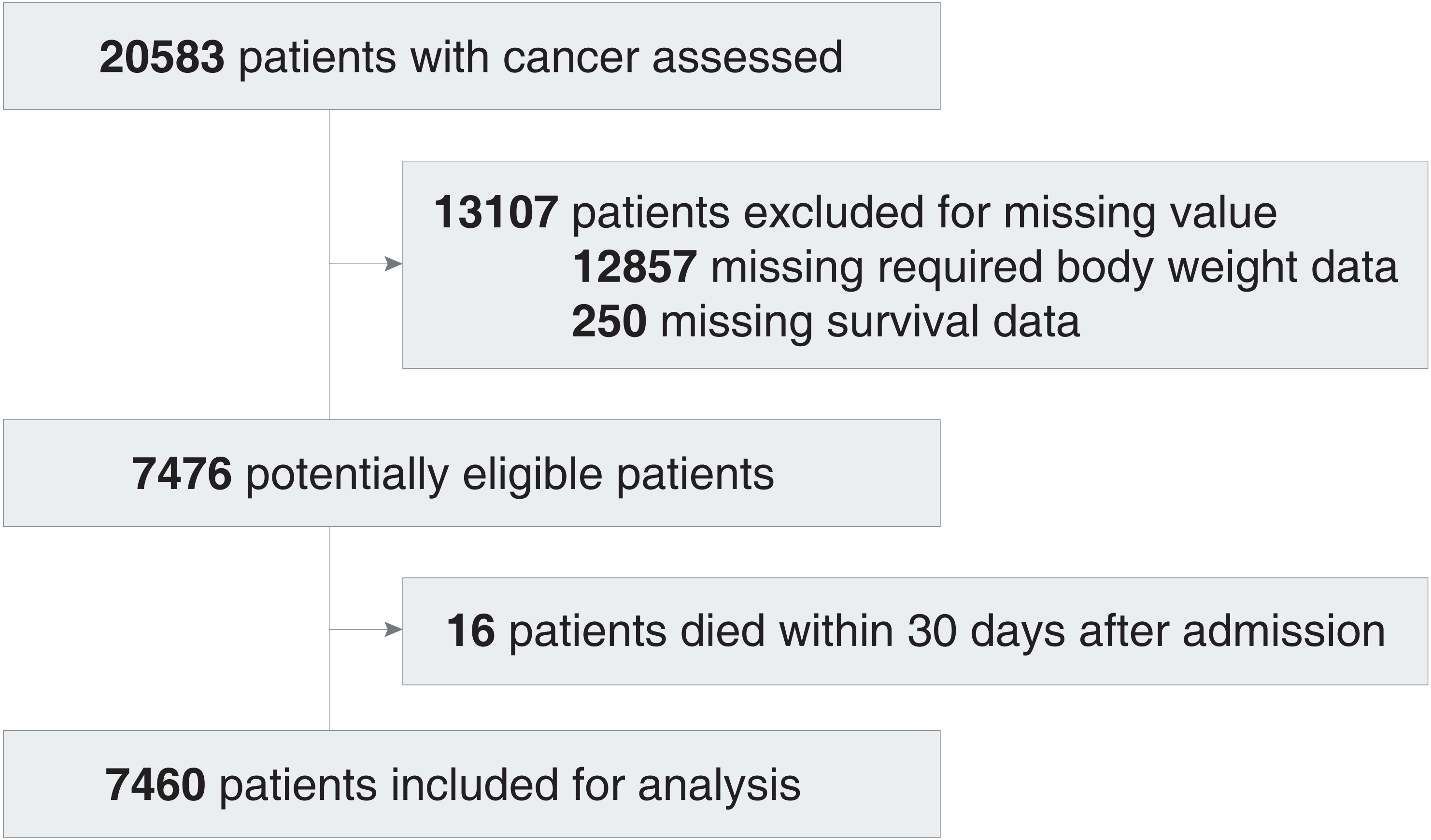
20583 patients with cancer assessed
13107 patients excluded for missing value
12857 missing required body weight data
250 missing survival data
7476 potentially eligible patients
16 patients died within 30 days after admission
7460 patients included for analysis
